# Half-Cell State of Charge Monitoring for Determination of Crossover in VRFB—Considerations and Results Concerning Crossover Direction and Amount

**DOI:** 10.3390/membranes11040232

**Published:** 2021-03-24

**Authors:** Theresa Haisch, Hyunjoon Ji, Lucas Holtz, Thorsten Struckmann, Claudia Weidlich

**Affiliations:** 1 DECHEMA Research Institute, Electrochemistry, Theodor-Heuss-Allee 25, 60486 Frankfurt am Main, Germany; theresa.haisch@dechema.de (T.H.); hyunjoonji85@gmail.com (H.J.); 2 Department of Mechanical Engineering and Production Management, Hamburg University of Applied Sciences, Berliner Tor 21, 20099 Hamburg, Germany; lucas-holtz@gmx.de (L.H.); thorsten.struckmann@haw-hamburg.de (T.S.)

**Keywords:** vanadium flow battery, crossover, state of charge, half-cell monitoring

## Abstract

Membranes play a crucial role in efficiency and longevity of flow batteries. Vanadium flow batteries suffer self-discharge and capacity fading due to crossover of electrolyte components through the membrane from one battery half-cell to the other. We consider the impact of vanadium species crossing ion exchange membranes on state of charge of the battery and we present a simple method to determine crossoverll open circuit potential measurements. State of s. State of charge for the negative and positive half-cell is simulated based on assumptions and simplifications for cation and anion exchange membranes and different crossover parameters. We introduce a crossover index “*Ind*${}_{Xovr}$” which enables the determination of crossover direction from state of charge data for the negative and positive half-cell and therewith identification of the half-cell in which predominant self-discharge occurs. Furthermore *Ind*${}_{Xovr}$ allows statements on crossover amount in dependence on state of operation. Simulated case studies are compared to experimental state of charge values estimated from half-cell potential measurements. Our results reveal that half-cell potential monitoring respectively half-cell SOC estimation, is a simple and suitable tool for the identification of crossover direction and relative amount of crossover in VFB.

## 1. Introduction

Flow batteries gain increasing interest for the storage of renewable energies due to their independent scalability of power and capacity. The energy is stored in electrolytes containing redox active species which are reduced and oxidized, respectively charged and discharged, during operation of the battery. The battery half-cells are separated by a membrane which prevents mixing of the redox active species of the negative half-cell (NHC) and positive half-cell (PHC) electrolytes, respectively avoids short circuit of the battery. The membrane is also needed as ionic conductor and should allow transfer of ions to assure charge balance. Therewith the membrane is crucial for the efficient operation of flow batteries and should provide high ionic conductivity combined with high selectivity. Development of membranes which fit these needs is still challenging and a lot of effort is spend on research on membranes for flow battery applications.

Amongst a variety of electrolyte chemistries vanadium flow batteries (VFB) are intensively studied and most common commercially applied [1,2,3]. VFB are equipped with vanadium electrolyte in both half-cells. Operation of the VFB, respectively charging and discharging, is connected to the following reactions in the NHC Equation (1) and in the PHC Equation (2).

(1) $$V^{3+}+e^{-}\overset{\mathrm{Charge}}{\underset{\mathrm{Discharge}}{⇌}}V^{2+}$$

(2) $$VO^{2+}+H_{2}O\overset{\mathrm{Charge}}{\underset{\mathrm{Discharge}}{⇌}}V{O_{2}}^{+}+2H^{+}+e^{-}$$

The aim of this work is to study the impact of vanadium ion crossover on the SOC of the VFB and to consider and determine crossover direction as well as relative amount of crossover using half-cell open circuit potential measurements which are compared to simplified simulated case studies.

Vanadium electrolytes, respectively active species of the same element, in both battery half-cells are advantageous because mixing of the half-cell electrolytes does not result in contamination of the electrolytes. Nevertheless undesirable crossover of electrolyte components, especially active vanadium species, has been observed for commercially applied membranes and results in self- discharge and capacity fading of the VFB [4,5,6].

The state of charge (SOC) of a battery is defined as the actual level of energy relative to its capacity and thus can be expressed by the ratio of active species to the overall vanadium concentration in each half-cell (Equations (3) and (4)).

(3) $$SOC_{NHC}=\frac{\left[V^{2+}\right]}{\left[V^{2+}\right]+\left[V^{3+}\right]}$$

(4) $$SOC_{PHC}=\frac{\left[VO_{2}^{+}\right]}{\left[VO_{2}^{+}\right]+\left[VO^{2+}\right]}$$

To ensure efficient charging and discharging of the battery, to avoid deep discharge and overcharge which might lead to degradation of the battery and also to develop effective operation strategies knowledge about the SOC is essential. Accurate and reliable online SOC determination is necessary to improve battery control and to achieve efficient long term operation of the system [7]. A number of methods exist for estimation of the SOC, including potential measurement, UV-Vis spectroscopy, density or conductivity [8,9,10,11,12,13].

To enable the determination of electrolyte imbalances in earlier stage as well as to improve the accuracy of SOC calculation, the open circuit potential (OCP) of the positive half-cell (PHC) and negative half-cell (NHC) can be monitored separately [10,11,14]. Half-cell SOC can be estimated from OCP measurements via Nernst equation for the PHC (Equation (5)) and PHC (Equation (6)) [12].

(5) $$\begin{array}{cc} OCP_{PHC}=\phi^{0+}-\frac{RT}{zF}\ln\left(\frac{\left(1-SOC\right)}{SOC\cdot {\left[H^{+}\right]}^{2}}\right) & \\ & \mathrm{with}\frac{\left(1-SOC\right)}{SOC}=\frac{\left[VO^{2+}\right]}{\left[VO_{2}^{+}\right]} \end{array}$$

(6) $$\begin{array}{cc} OCP_{NHC}=\phi^{0-}-\frac{RT}{zF}\ln\left(\frac{SOC}{\left(1-SOC\right)}\right) & \\ & \mathrm{with}\frac{SOC}{\left(1-SOC\right)}=\frac{\left[V^{2+}\right]}{\left[V^{3+}\right]} \end{array}$$

$\phi^{0}$ is standard electrode potential; *R* is universal gas constant (*R* = 8.3145 J K${}^{-1}$ mol${}^{-1}$; *T* is temperature (in K); *z* is number of electrons transferred in half-cell reaction; *F* is Faraday constant (*F* = 96,485 J mol${}^{-1}$).

SOC estimation for the individual half-cells from OCP measurements is suitable for the investigation of crossover characteristics [10,11,14]. Based on the monitoring of the individual half-cell SOC, it is also possible to obtain additional information about the character of the crossover regarding direction, relative amount and state of operation.

### Crossover Processes through Ion Exchange Membranes

Research and effort have been focused on development and optimization of ion exchange membranes to provide the desired combination of high conductivity and selectivity [15,16,17,18,19,20]. Besides, also other types of membranes, for example amphoteric or nanoporous membranes, have been investigated [21,22,23,24]. Nevertheless the membranes exhibit a certain permeability for vanadium ions. The relevant reactions in VFB caused by crossing of vanadium species from one half-cell to another are summarized following. Crossover of vanadium ions through the membrane from the NHC to the PHC results in capacity fading at the NHC and self-discharge of the PHC electrolyte [4,25].

(7) $$V^{2+}+2V{O_{2}}^{+}+2H^{+}\to 3VO^{2+}+H_{2}O$$

(8) $$V^{3+}+V{O_{2}}^{+}\to 2VO^{2+}$$

Crossover from the PHC to the NHC leads to capacity loss in the PHC and self-discharge in the NHC.

(9) $$VO^{2+}+V^{2+}+2H^{+}\to 2V^{3+}+H_{2}O$$

(10) $$V{O_{2}}^{+}+V^{2+}+4H^{+}\to 3V^{3+}+2H_{2}O$$

To characterize ion exchange membranes concerning their applicability in VFB, crossover processes have extensively been studied and are summed up below. Driving forces for electrolyte components crossing the membrane are diffusion, migration and osmotic pressure as well as electroosmotic convection [26,27,28,29]. The net direction of the crossover is composed of the contributions of the individual transport modes and is accordingly affected by various factors.

Crossover driven by migration and electroosmosis is supposed to be proportional to current density and independent on membrane thickness, whereas diffusion is inversely proportional to membrane thickness and independent on current density [4,28,29]. Which ions cross the membrane depends on the type of membrane used in the VFB as well as on membrane material, state of operation and SOC of the electrolyte [15,19,24,28,30,31]. Cation exchange membranes (CEM) allow the exchange of cations, the charge balance is maintained via protons. Therefore CEM have a certain permeability for vanadium cations and it is challenging to prevent crossover. Using anion exchange membranes (AEM), hydrogen sulfates cross the membrane to maintain charge balance and vanadium ions should be excluded. AEM suffer from a lower ionic conductivity, but show significantly lower crossover rates because of the Donnan exclusion effect [19,32]. However, crossover of vanadium ions through AEM has been observed and the presence of various negatively charged vanadium-sulfate complexes at high acid concentrations, which are able to diffuse through an AEM are reported (11) [24,31,33].

(11) $$VO_{2}SO_{4}^{-}+2V^{2+}+5H^{+}\to 3V^{3+}+2H_{2}O+HSO_{4}^{-}$$

**Figure 1:**
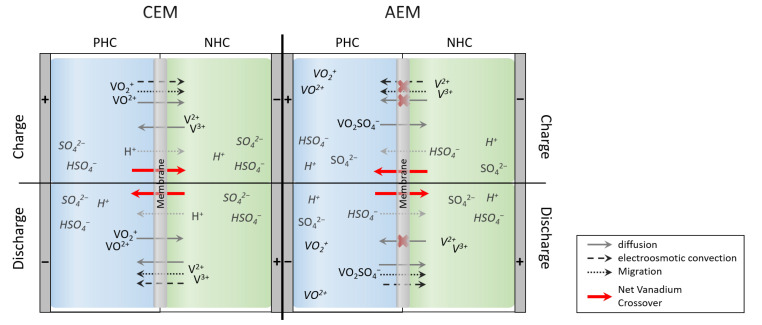
Simplified scheme for vanadium crossover processes in dependence on membrane type (cation and anion exchange membrane) and state of operation (charge and discharge). Transfer of ions for charge balance is included, transfer of water is not considered.

Based on the described crossover processes, the scheme in Figure 1 is considering the crossover of ions in dependence on ion exchange membrane type and state of operation (charge or discharge) of the battery in a simplified manner; water transfer is not considered. The transfer of water is caused by osmotic pressure, by the transfer of vanadium ions carrying water molecules bound within the hydrate shell and also by the charge balancing ions (protons, hydrogen sulfate) [25,34,35,36]. Ions with a high charge carry larger amounts of water molecules. Since the charge of vanadium ions as well as osmotic pressure depend on the SOC, the transfer of water also depends on SOC [37,38].

Crossover induced imbalance between the electrolytes of the NHC and the PHC accumulates during operation of VFB but is reversible and can be overturned by remixing or rebalancing of the vanadium electrolyte [9,39,40,41]. Knowledge about crossover processes is essential for efficient longterm operation and successful remixing and rebalancing procedures of the electrolyte. Furthermore, identification and distinction of reversible crossover processes from irreversible degradation of the VFB allow reliable state of health (SOH) estimation and enable life time prolongation. Additional to crossover processes other side reactions of the electrolyte, e.g., vanadium precipitation, oxygen or hydrogen evolution, oxidation of NHC electrolyte, may occur during operation of a VFB and have an irreversible impact on capacity of the VFB [42].

## 2. Materials and Methods

To validate the considerations and assumptions on crossover direction and relative amount, we tested membranes with different ion exchange properties and thickness. Using also different battery cells and charge discharge parameters we wanted to show the comparability of the results for a crossover index under different conditions.

The charge and discharge experiments were performed in a VFB single cell (DECHEMA machine shop or Micro Flow Cell, ElectroCell, Tarm, Danmark). The membrane (FUMATECH BWT GmbH, Bietigheim-Bissingen, Germany) was placed between two electrode felts (SIGRACELL^®^ GFD 4.6, SGL Carbon, Wiesbaden, Germany). The connection to the potentiostat (Gamry Instruments, Pennsylvania, USA; Greenlight Innovation, Rheinau, Germany or Biologic, Muenster, Germany) was established via graphite bipolar (Schunk Carbon Technology, Heuchelheim, Germany) and copper plates. For all measurements, a vanadium electrolyte is used, which initially consists of 50% V${}^{3+}$ and 50% VO${}^{2+}$ (GfE Metalle und Materialien GmbH, Nuernberg, Germany). During a conditioning preceding the charge and discharge cycles, the existing VO${}^{2+}$ is reduced to V${}^{3+}$ in the NHC, while the V${}^{3+}$ in the PHC is oxidized to VO${}^{2+}$. So at the end of the conditioning, there is 100% V${}^{3+}$ in the NHC and 100% VO${}^{2+}$ in the PHC, which is equal to a SOC of 0% in both half-cells. Besides vanadium species, the electrolyte mainly contains sulfuric acid and low concentrations of additives (e.g., phosphoric acid). The electrolyte was pumped through the system together with nitrogen or argon to exclude oxidation by oxygen. Membranes and parameters can be found in Table 1. An auxiliary flow cell (DECHEMA Machine Shop) in which the open circuit potential (OCP) of the respective half-cell is recorded during cycling was connected at the cell outlets on each side. Each flow cell is equipped with a Hg/Hg${}_{2}$SO${}_{4}$ reference electrode (HgE 11-S, Sensortechnik Meinsberg Xylem Analytics GmbH & Co. KG., Meinsberg, Germany) and a glassy carbon rod (SIGRADUR^®^G, Hochtemperatur-Werkstoffe GmbH, 2 mm diameter, Thierhaupten, Germany). Further details can be found in previous publications [10,11,14].

## 3. Impact of Vanadium Crossover on State of Charge: Case Studies

Crossover processes for different types of membranes as well as for degraded membranes and in dependence on the state of operation of the VFB have extensively been studied and complex models have been developed to unravel and predict the transport methods [4,15,24,28,31,35,36,43,44,45,46]. The dependence of crossover on various parameters and conditions complicates the characterization and comparison of different membranes concerning their applicability for VFB. Therefore, our focus is not on sophisticated modeling. Rather it is to develop a simple method using experimental data to determine crossover characteristics, namely direction and state of operation. In this section, we consider crossover and the associated impact on the SOC and describe our approach to theoretical calculations. Based on different case studies we analyze and compare representative case studies and discuss the calculated SOC in order to obtain information about crossover characteristics and compare our findings to experimental data. The following specifications provide the basis for our considerations:

- Net crossover of vanadium ions results in self-discharge of one half-cell of the VFB predominantly; which half-cell is affected most depends on the type and material of the membrane and state of operation.
- Amount of vanadium ions crossing depends on current density and membrane thickness.
- Crossover of vanadium ions results from different processes (diffusion, migration and electroosmotic convection) which may coincide or counteract.

### 3.1. Vanadium Crossover: Assumptions

For the theoretical determination of the SOC, concentration profiles of the four vanadium species in the respective half-cells are simulated for different case studies. A total concentration of 1.6 M vanadium per half-cell is assumed as the starting value for all case studies. The conditioning step, during which crossover can already take place, is not taken into account. For the calculation of the individual vanadium concentration profiles during cycling of the battery and the resulting SOC, further assumptions are made.

- Only V${}^{2+}$ and VO${}_{2}^{+}$ ions are assumed to cross the membranes since the resulting self discharge is more explicit.
- Each vanadium ion crossing the membrane reacts stochiometrically in the other half-cell, other side reactions or crossing of other electrolyte components, e.g., H${}^{+}$ or HSO${}_{4}^{-}$ are neglected.
- The amount of vanadium crossover is defined and kept constant for each cycle within the calculated charge-discharge (cd) sequences if not otherwise claimed.
- For charging and discharging the VFB, it is assumed that a maximum of 100% and minimum of 0% is achievable.
- No distinction is made between current density or thickness dependent crossover respectively between migration, electroosmotic convection and diffusion driven crossover.
- Water transport is neglected and a constant volume in the half-cells is assumed.

Based on the resulting concentration profiles, the SOC of the respective half-cells can be calculated using Equations (3) and (4).

In order to consider the influence of crossover on the SOC, we take into account ion exchange membrane type (CEM or AEM), vanadium species which is predominantly crossing (V${}^{2+}$ or VO${}_{2}^{+}$), crossover direction (NHC to PHC and vice versa) and state of operation (charge or discharge). Also a defined amount of ions is postulated to cross the membrane [45]. These parameters are combined in order to create several case studies which are listed in Table 2.

CEM are predominantly crossed by V${}^{2+}$ ions (case I) but also VO${}_{2}^{+}$ ions might have an decisive impact (case II) [2,30,45]. Crossover is supposed to occur in the same direction during charge and discharge if diffusion is the predominant driving force for crossover (case I). For migration and electroosmotic convection dominating as transport mechanisms, crossover direction changes in dependence on charge and discharge (case II). AEM can be crossed by vanadiumsulfate anions (e.g., VO${}_{2}$SO${}_{4}^{-}$) [17,19,33,37]. In case III predominant crossover of VO${}_{2}$SO${}_{4}^{-}$ is assumed during charging whereas in case IV predominant crossover of VO${}_{2}$SO${}_{4}^{-}$ is assumed during discharging.

For cases I-IV, concentration profiles of all vanadium species in both half-cells are calculated over 10 cycles (see Figure S1 in SI). According to Equations (3) and (4), the SOC of the individual half-cells can be calculated directly from the concentrations.

### 3.2. Simulation of Crossover Direction

**Figure 2:**
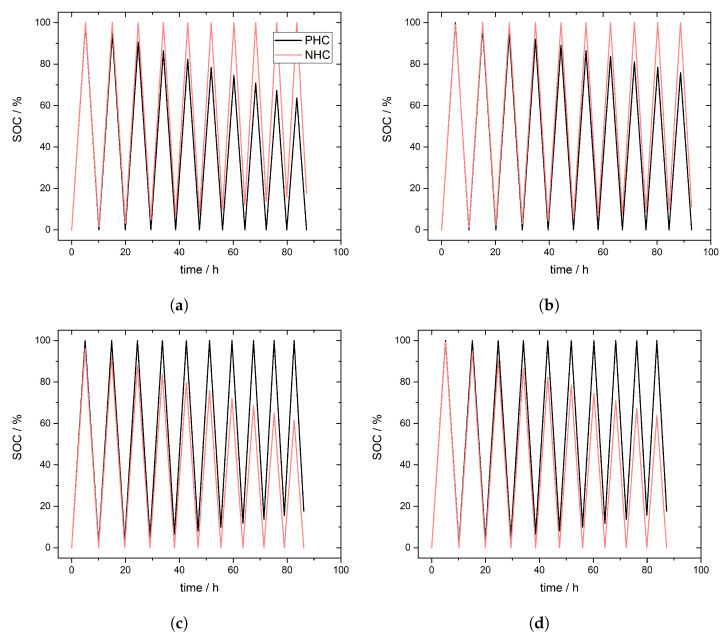
Simulated SOC in the positive half-cell (PHC) and negative half-cell (NHC) for 10 charge and discharge cycles according to parameters in Table 2. (**a**) Case I (CEM), (**b**) Case II (CEM) (**c**) Case III (AEM) and (**d**) Case IV (AEM).

Figure 2a depicts the simulated SOC in the PHC and the NHC for case I. The crossover direction is set to be independent on the state of operation, V${}^{2+}$ ions are predominantly crossing from NHC to PHC both during charge and discharge. Crossover is assumed to be stronger during discharging due to additional migration and electroosmotic convection of V${}^{2+}$. In accordance to Equation (7) self-discharge occurs in the PHC and the SOC for the charged PHC is decreasing from cycle to cycle. The self-discharge process during charging works opposed to the charge process. Already formed VO${}_{2}^{+}$-ions are reduced to VO${}^{2+}$-ions and are theoretically available for further charging. However, since one V${}^{2+}$ is formed per VO${}_{2}^{+}$ during charging, the NHC is fully charged while VO${}^{2+}$-ions remain in the PHC and the SOC for the charged PHC is decreasing from cycle to cycle. During discharging the lowered SOC of the PHC defines the completion of the process. Due to the self-discharge during charging a lower amount of VO${}_{2}^{+}$-ions (PHC) is present compared to V${}^{2+}$-ions (NHC). In addition, the self-discharge during discharging promotes the discharge process in the PHC. Therewith a part of V${}^{2+}$-ions remain in the NHC and prevent a complete discharge, so the SOC of the discharged NHC is increasing from cycle to cycle. Overall, due to the crossover, the SOC range for both half-cells decreases from cycle to cycle.

Figure 2b depicts the SOC in the PHC and the NHC for case II. The crossover direction is inversed between charging and discharging [4,45]. As in case I in accordance to Equation (7) self-discharge occurs in the PHC during discharging. But in contrary to case I also VO${}_{2}^{+}$-ions are involved in crossover processes and dominates during charging crossing from PHC to NHC. In accordance to Equation (10) self-discharge occurs in the NHC. The crossover during discharging is assumed to be higher than during charging due to additional migration and electroosmotic convection of V${}^{2+}$. Both crossover induced self-discharge processes partly neutralize each other. As a result, the SOC range is decreasing less compared to case I. This is in agreement with results reported on the reversion of crossover direction leading to a lower “net” crossover [28].

Figure 2c,d show simulated SOC in PHC and NHC for cases III and IV. The crossover direction is independent on the state of operation, VO${}_{2}^{+}$ (as VO${}_{2}$SO${}_{4}^{-}$) ions are crossing from PHC to NHC. Figure 2c refers to predominant crossover during charging whereas Figure 2d depicts predominant crossover during discharging. In both cases self-discharge occurs in accordance to Equation (10) in the NHC and the SOC for the charged NHC is decreasing from cycle to cycle.

**Figure 3:**
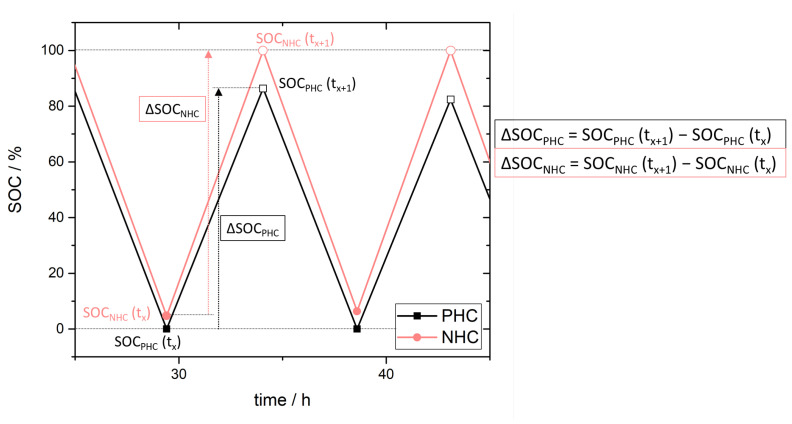
Scheme for the determination of crossover index *Ind*${}_{Xovr}$ from state of charge (SOC) in the positive half-cell (PHC) and negative half-cell (NHC).

Based on the above considerations and assumptions we suggest a simple approach to estimate net crossover direction. Using the half-cell SOC data, comparison of the SOC range for the PHC and NHC enables an unambiguous assignment of the net crossover direction. Defining a SOC progress ΔSOC for the PHC (Equation (12)) and NHC (Equation (13)) as the SOC difference between two points in time, respectively the maximum and minimum SOC of a charge or discharge half-cycle (Figure 3), we put ΔSOC for PHC and NHC in relation to one another. This ratio, ΔSOC${}_{PHC}$/ΔSOC${}_{NHC}$, shall describe a crossover index for membranes used in VRFB and is named *Ind*${}_{Xovr}$.

(12) $$\Delta SOC_{PHC}=SOC_{PHC}\left(t_{x+1}\right)-SOC_{PHC}\left(t_{x}\right)$$

(13) $$\Delta SOC_{NHC}=SOC_{NHC}\left(t_{x+1}\right)-SOC_{NHC}\left(t_{x}\right)$$

(14) $$Ind_{Xovr}=\frac{\Delta SOC_{PHC}}{\Delta SOC_{NHC}}$$

For predominant crossover processes from NHC to PHC (mainly occurring using CEM) the crossover impact on the SOC of the PHC is stronger due to self-discharge. Therewith the SOC range (ΔSOC) for the charged and discharged PHC is decreased stronger than for the NHC and comparison of SOC ranges for PHC and NHC by putting them into relation as a ratio results in values < 1 (Equation (14)). For crossover processes from PHC to NHC (majorly occurring using AEM) the NHC suffers self-discharge. Hereby the crossover impact on the SOC range of the NHC is stronger and relation of ΔSOC for PHC and NHC results in values > 1 (Equation (14)). Since SOC of the NHC and PHC is directly measurable as redox potential of the NHC and PHC electrolyte, estimation of ΔSOC${}_{PHC}$/ΔSOC${}_{NHC}$ is quite simple to realize [12].

**Figure 4:**
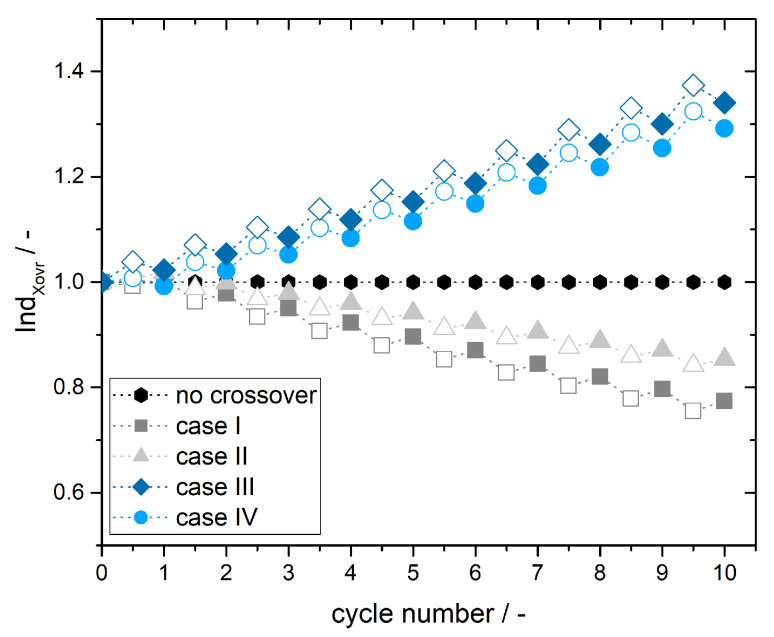
Simulated crossover index *Ind*${}_{Xovr}$ for cases I-IV. Case I and II refers to vanadium species crossing from the negative half-cell (NHC) to the positive half-cell (PHC), Case III and IV considers crossover from PHC to NHC. According to cycle numbers full numbers display a discharge half-cycle (filled icons) and half numbers (x,5) display a charge half-cycle (empty icons).

Figure 4 shows the simulated *Ind*${}_{Xovr}$ over 10 cycles for cases I-IV. As described by Equation (14) *Ind*${}_{Xovr}$ is larger than 1 for crossover from PHC to NHC and lower than 1 for crossover from NHC to PHC. If no crossover occurs, the ratio would be constant for all cycles (*Ind*${}_{Xovr}$ = 1). Values for *Ind*${}_{Xovr}$ are steadily increasing with increasing number of cycles in case of crossover from PHC to NHC (cases III and IV) and steadily decreasing in case of crossover from NHC to PHC (cases I and II). This is due to crossover progressing from cycle to cycle resulting in accumulation of discharged vanadium species in the battery half-cells. Since vanadium crossover is assumed to progress linear from cycle to cycle no exponential behavior or stagnation is displayed.

*Ind*${}_{Xovr}$ is determined and plotted in Figure 4 for the charge as well as discharge half-cycles and depicts a steeper slope for the charge half-cycles than for the discharge half-cycles. This can be found for crossover from PHC to NHC (cases III and IV) with a positive slope and for crossover from NHC to PHC (cases I and II) with a negative slope and is caused by the different impact of crossover, respectively self-discharge, on the charge and discharge process. Crossover during charging impedes the charge process whereas the discharge process is promoted by crossover due to additional self-discharge. Therewith for the charge process the ΔSOC of the half-cell which suffers self-discharge is smaller and *Ind*${}_{Xovr}$ deviates stronger from 1 than for the discharge process.

In addition to the determination of the crossover direction, also information on the relative amount of vanadium ions can be considered. Comparison of case I and II depicts a steeper slope for *Ind*${}_{Xovr}$ with increasing charge and discharge cycles. This is due to a larger amount of V${}^{2+}$ ions crossing during charging and discharging from NHC to PHC in case I. Whereas in case II V${}^{2+}$ ions are crossing only during discharging and therewith a smaller total amount of V${}^{2+}$ ions is transferred to the PHC. Furthermore the crossover is reversed during charging by transfer of VO${}_{2}^{+}$ from the PHC to the NHC and the crossover from NHC to PHC is partly neutralized.

Comparison of cases III and IV depicts higher *Ind*${}_{Xovr}$ and a slightly steeper slope for case III despite the amount of VO${}_{2}$SO${}_{4}^{-}$ crossing during a full cycle (charge and discharge cycle) is the same as for case IV. The higher *Ind*${}_{Xovr}$ is due to the higher amount of VO${}_{2}$SO${}_{4}^{-}$ ions crossing during the initial charge step for case III. Since self-discharge accumulates for the case with the higher crossover amount during the initial half cycle *Ind*${}_{Xovr}$ is also higher for the following cycles.

### 3.3. Simulation of Crossover Amount

**Figure 5:**
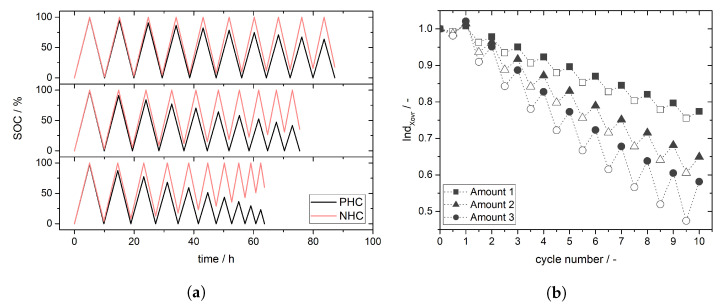
(**a**) Simulated SOC curves for case I and different crossover quantities. The amount of vanadium ions crossing increases from top to bottom (Amount 1 < Amount 2 < Amount 3). (**b**) Crossover index *Ind*${}_{Xovr}$ calculated from the data in (**a**) According to cycle numbers full numbers display a discharge half-cycle (filled icons) and half numbers (x,5) display a charge half-cycle (empty icons).

The amount of vanadium ions crossing the membrane depends on membrane thickness and current density. Simplified, higher current densities as well as thinner membranes majorly promote stronger crossover and thereby higher capacity fading and stronger self-discharge. Figure 5a shows simulated SOC for case I with three different amounts of vanadium ions crossing from NHC to PHC (variations of case I, see Table S1 in SI). It is obvious that increasing amount of vanadium ions crossing through the membrane leads to shortening of charge and discharge cycles as well as decreasing SOC range (decreasing of maximum SOC and increasing minimum SOC) [35].

Figure 5b shows the crossover index *Ind*${}_{Xovr}$ for the different crossover amounts. A larger amount of vanadium ions crossing the membrane from NHC to PHC (*Ind*${}_{Xovr}$ < 1) leads to a stronger deviation of *Ind*${}_{Xovr}$ from 1 and steeper slope. Also the difference between *Ind*${}_{Xovr}$ for a charge half-cycle and *Ind*${}_{Xovr}$ for a discharge half-cycle depends on the amount of crossover; the difference is significantly larger for higher crossover amount and is increasing from cycle to cycle due to accumulation of discharged V species in the PHC (VO${}^{2+}$). As stated above the difference between *Ind*${}_{Xovr}$ for charge and discharge half-cycle is caused by the different impact of crossover on SOC during the charge and discharge process. Crossover during charging impedes the charge process whereas the discharge process is promoted by crossover due to additional self-discharge.

## 4. Experimental Results and Discussion: Determination of Crossover Direction and Amount

**Figure 6:**
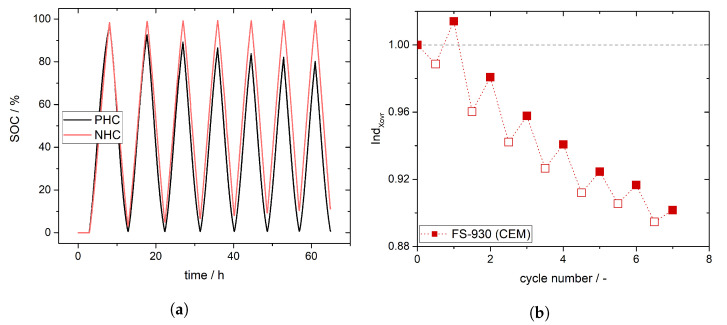
(**a**) State of charge (SOC) estimated from redox-potential measurements in the positive half-cell (PHC) and negative half-cell (NHC), battery cell with cation exchange membrane (FS-930), parameters for charge and discharge cycles in Table 1. (**b**) Resulting crossover index *Ind*${}_{Xovr}$ for the SOC data in (**a**). According to cycle numbers full numbers display a discharge half-cycle (filled icons) and half numbers (x,5) display a charge half-cycle (empty icons).

To reveal the applicability of the crossover index, experimental data for different membranes have been collected under various conditions (Table 1). Figure 6a represents the SOC calculated from half-cell OCP measurement in the NHC and PHC of a battery cell with a CEM (FS-930). The progression of the SOC for NHC and PHC depicts the predicted predominant crossover from the NHC to PHC in accordance to case I and II (Figure 2a,b). The crossover index *Ind*${}_{Xovr}$< 1 also testifies the expected direction of net crossover (Figure 6b). In contrary to the case studies in which the simulated crossover of vanadium ions has been assumed to be constant, (Figure 4), the difference between *Ind*${}_{Xovr}$ for a charge half cycle and a discharge half cycle is not increasing from cycle to cycle but rather significantly decreasing. That means that crossover is decreasing from cycle to cycle. This is in accordance to the observed crossover behavior during application of ion exchange membranes in VFB; significant crossover of vanadium ions crossing can be observed for the first few charge and discharge cycles but stabilizes and decreases during cycling.

**Figure 7:**
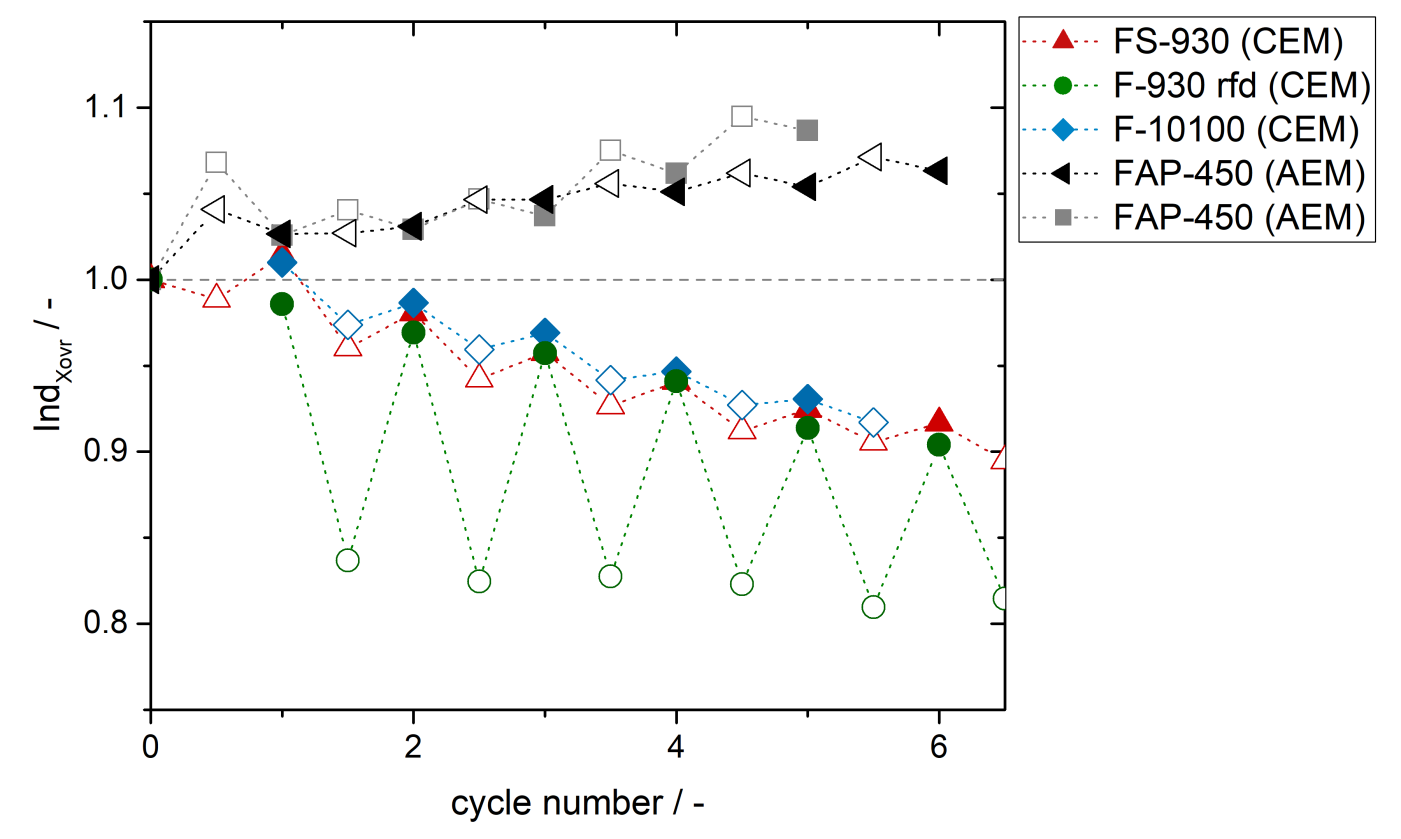
Crossover index *Ind*${}_{Xovr}$ for state of charge (SOC) estimation from redox-potential measurements in the positive half-cell and negative half-cell, battery cell with membranes and parameters for charge and discharge cycles in Table 1. According to cycle numbers full numbers display a discharge half-cycle (filled icons) and half numbers (x,5) display a charge half-cycle (empty icons).

The results for *Ind*${}_{Xovr}$ of all investigated membranes are summarized in Figure 7 (*Ind*${}_{Xovr}$ = 1 means no crossover). *Ind*${}_{Xovr}$ for the tested AEM are higher than 1 as predicted due to vanadium ions crossing the membrane from PHC to NHC. *Ind*${}_{Xovr}$ for experiment FAP-450 (AEM 1) and therewith crossover is slightly higher than for experiment FAP-450 (AEM 2) due to a higher amount of vanadium ions crossing driven by higher current densities.

*Ind*${}_{Xovr}$ for the tested CEM are smaller than 1 and *Ind*${}_{Xovr}$ for the thinner membrane (FS-930) is lower respectively deviates stronger from 1 than for the thicker membrane (F-10100) due to a higher amount of vanadium ions crossing the thinner membrane. Also the difference between *Ind*${}_{Xovr}$ for the charge and discharge half-cycles is smaller for the thicker membrane since less crossover occurs.

With membrane F-930 rfd the *Ind*${}_{Xovr}$ for the charge half-cycles differ very strong from *Ind*${}_{Xovr}$ for the discharge half-cycles. The difference is decreasing from cycle to cycle as described for the FS-930 membrane (Figure 6b). Despite comparable membrane thickness the difference between *Ind*${}_{Xovr}$ for the charge half-cycles of the FS-930 and F-930 rfd is significant whereas the differences between the *Ind*${}_{Xovr}$ for the discharge half-cycles for the FS-930 and F-930 rfd are only small. This might be caused by different material properties (short side chains in FS-930) and also different ion exchange properties; e.g., crossing of VO${}_{2}^{+}$, concurrent with comparable thickness of the membranes.

Comparison of experimental results to simulated case studies reveals applicability of *Ind*${}_{Xovr}$. Crossover direction can be identified using half-cell SOC estimation data and also the relative amount of crossover can be determined. Since the simulation of case studies aimed rather to study the feasibility of *Ind*${}_{Xovr}$ than to develop a model which fits the results measured, *Ind*${}_{Xovr}$ from simulated and measured half-cell SOC are not correlated or compared quantitatively.

## 5. Conclusions

Crossover trough membranes employed in VFB is complex and depends on various parameters. Several detailed models have already been developed to picture and predict the crossover processes and their impact on the battery electrolyte. The dependence of crossover on various conditions makes it difficult to characterize and compare different membranes concerning their ability to diminish crossover and applicability in VFB.

To simplify the evaluation of membranes and to enhance the comparability of different membranes concerning crossover of vanadium ions we introduce a crossover index *Ind*${}_{Xovr}$. This crossover index is a ratio of a state of charge (SOC) range ΔSOC for the positive half-cell (PHC) and the negative half-cell (NHC) due to vanadium ions passing the membrane during the charge or discharge process (ΔSOC${}_{PHC}$/ΔSOC${}_{NHC}$) and gives information about the direction and relative amount of crossover.

Considerations of the crossover index concerning applicability for the determination of crossover direction and amount based on several case studies are verified by experimental results. The results coincide with the case studies and reveal the applicability of the crossover index for comparison of membranes concerning crossover characteristics in VFB as vanadium crossover direction and relative amount. Furthermore comparison of several membranes tested under various conditions is possible without further adaption or conversion of parameters and results.

## Figures and Tables

**Table 1:** Overview of the cation exchange membranes (CEM)/anion exchange membranes (AEM) and parameters used for experiments.

Membrane	Thickness	Electrolyte	Electrode	Potential	Current	Electrolyte
		Volume	Area	Limits	Density	Flow
	(m)	(mL)	(cm${}^{2}$)	(V)	(mA cm${}^{-2}$)	(L h${}^{-1}$)
CEM						
FS-930	30	60	10	0.8–1.65	50	1.5
F-930 rfd	30	100	10	0.8–1.70	60	3
F-10100	100	100	10	0.8–1.70	35	3
AEM						
FAP-450 1	50	100	10	0.8–1.70	60	3
FAP-450 2	50	500	40	0.8–1.65	50	3

**Table 2:** Crossover case studies in dependence on membrane type, crossover direction, amount and state of operation.

	Case I	Case II	Case III	Case IV
Membrane Type	CEM	CEM	AEM	AEM
Crossover charge during
Direction	NHC→PHC	PHC→NHC	PHC→NHC	PHC→NHC
Predominant V-species	V${}^{2+}$	VO${}_{2}^{+}$	VO${}_{2}$SO${}_{4}^{-}$	VO${}_{2}$SO${}_{4}^{-}$
Amount	0.004 M	0.004 M	0.02 M	0.004 M
Crossover discharge during
Direction	NHC→PHC	NHC→PHC	PHC→NHC	PHC→NHC
Predominant V-species	V${}^{2+}$	V${}^{2+}$	VO${}_{2}$SO${}_{4}^{-}$	VO${}_{2}$SO${}_{4}^{-}$
Amount	0.02 M [45]	0.02 M	0.004 M	0.02 M
Predominant during	discharge [28]	discharge [28]	charge	discharge

## Data Availability

Data is contained within the article or supplementary material.

## Supplementary Materials

The following are available online at https://www.mdpi.com/2077-0375/11/4/232/s1, Figure S1: Simulated concentrations profiles of vanadium ions in the positive half-cell (PHC) and negative half-cell (NHC) for 10 charge and discharge cycles. (a) Case I (CEM), (b) Case II (CEM) (c) Case III (AEM) and (d) Case IV (AEM)., Table S1: Overview of variations of case I.
