# Supplementary material for: Half-Cell State of Charge Monitoring for Determination of Crossover in VRFB—Considerations and Results Concerning Crossover Direction and Amount
Source: Membranes (Basel). 2021 Mar 24;11(4):232. doi: 10.3390/membranes11040232 (PMC8064305; doi:10.3390/membranes11040232)
Supplement: Supplementary file 1 [file membranes-11-00232-s001.pdf]

membranes

Article

Half-cell state of charge monitoring for determination of crossover in VRFB - Considerations and results concerning crossover direction and amount

Electronic Supplementary Information

Theresa Haisch [1], Hyunjoon Ji[1], Lukas Holtz [2], Thorsten Struckmann [2], Simon Ressel, Tomas Klicpera [2] and Claudia Weidlich [1,*]

1 DECHEMA Research Institute, Electrochemistry, Theodor-Heuss-Allee 25, 60486 Frankfurt am Main, Germany
2 Hamburg University of Applied Sciences, Department of Mechanical Engineering and Production Management, Berliner Tor 21, 20099 Hamburg, Germany
* Correspondence: claudia.weidlich@dechema.de; Tel.: +49-(0)69-7564-633

This PDF file includes:

Figure S1
Table S1

check for updates

Citation: Haisch, T., Ji, H., Holtz, L., Ressel, S., Struckmann, T., Weidlich, C. Half-cell state of charge monitoring for determination of crossover in VRFB - Considerations and results concerning crossover direction and amount. *Membranes* **2021**, *11*, 232. https://doi.org/10.3390/membranes11040232

Received: 19 February 2021
Accepted: 16 March 2021
Published: 24 March 2021

Publisher's Note: MDPI stays neutral with regard to jurisdictional claims in published maps and institutional affiliations.

Copyright: © 2021 by the authors. Licensee MDPI, Basel, Switzerland. This article is an open access article distributed under the terms and conditions of the Creative Commons Attribution (CC BY) license (https://creativecommons.org/licenses/by/4.0/).

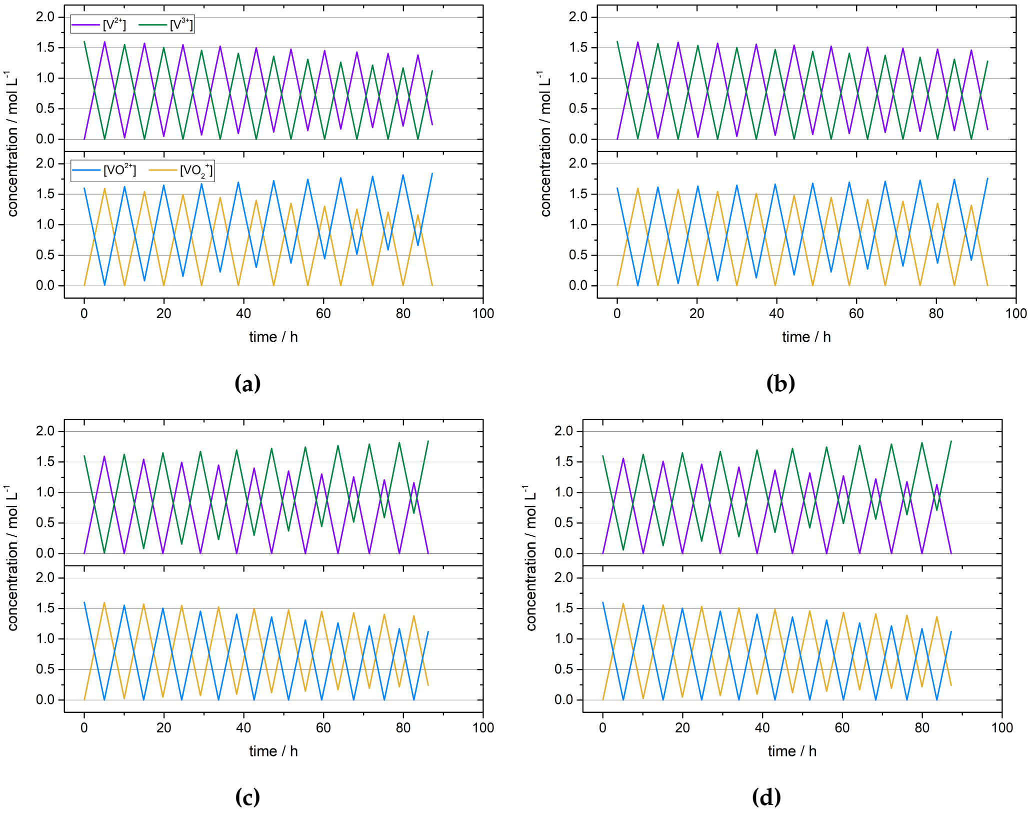

Figure S1. Simulated concentrations profiles of vanadium ions in the positive half-cell (PHC) and negative half-cell (NHC) for 10 charge and discharge cycles. a) Case I (CEM), b) Case II (CEM) c) Case III (AEM) and d) Case IV (AEM).

Table S1. Crossover case studies in dependence on membrane type, crossover direction, amount and state of operation.

	**Amount I**	**Amount II**	**Amount III**
Charge			
Crossover amount	0.004 M	0.007 M	0.01 M
Discharge			
Crossover amount	0.02 M	0.035 M	0.05 M

Figure S1 show the simulated concentration profiles for cases I-IV. The SOC has been calculated based on these values.

Table S1 gives the values for crossover during charging and discharging which are assumed for the investigation of the crossover amount. Amount I refers to Case I (see Table 1), the others are variations of case 1 and differ only in the amount.
